# Pro-Angiogenic and Pro-Inflammatory Regulation by lncRNA MCM3AP-AS1-Mediated Upregulation of DPP4 in Clear Cell Renal Cell Carcinoma

**DOI:** 10.3389/fonc.2020.00705

**Published:** 2020-06-23

**Authors:** Ling Qiu, Yan Ma, Yanming Yang, Xiaojun Ren, Dongzhou Wang, Xiaojing Jia

**Affiliations:** Department of Tumor Radiotherapy, The Second Hospital of Jilin University, Changchun, China

**Keywords:** clear cell renal cell carcinoma, long non-coding RNA, MCM3AP-AS1, E2F transcription factor 1, dipeptidyl peptidase-4

## Abstract

Clear cell renal cell carcinoma (ccRCC) represents the most common type of renal cell carcinoma (RCC) in adults, in addition to the worst prognosis among the common epithelial kidney tumors. Inflammation and angiogenesis seem to potentiate tumor growth and metastasis of the malignancy. The current study explored the contributions of the lncRNA MCM3AP-AS1 in tumor-associated inflammation and angiogenesis in ccRCC with a specific focus on its transcriptional regulation and its interactions with transcription factor E2F1 and DPP4. Tumor tissues and matched adjacent non-tumor tissues were collected from 78 ccRCC patients. Methylation-specific PCR and ChIP assays were applied to detect the methylation at the promoter region of MCM3AP-AS1. Dual-luciferase reporter assay, RIP, RNA pull-down, and ChIP assays were employed to confirm the interactions between MCM3AP-AS1, E2F1, and DPP4. Nude mice were subcutaneously xenografted with human ccRCC cells. Cell proliferation was evaluated by CCK-8 assays and EDU staining in ccRCC cells *in vitro* and by immunohistochemical staining of Ki67 *in vivo*. Inflammation was examined by detecting the secretion of pro-inflammatory cytokines (TNF-α, IL-1β, and IL-6). Pro-angiogenic ability of ccRCC cells was assessed by the co-culture with human umbilical vein endothelial cells (HUVEC) *in vitro* and by microvessel density (MVD) measurements and angiogenesis in the chicken chorioallantoic membrane. MCM3AP-AS1 was highly-expressed in ccRCC and associated with poor patient survival. Demethylation of MCM3AP-AS1 was noted in ccRCC tissues and cells. Over-expression of MCM3AP-AS1 enhanced cell proliferation, the release of pro-inflammatory cytokines, and the tube formation of HUVECs in cultured human Caki-1 and 786-O cells. MCM3AP-AS1 was shown to enhance the E2F1 enrichment at the DPP4 promoter, to further increase the expression of DPP4. Knockdown of DPP4 could abate pro-angiogenic and pro-inflammatory abilities of MCM3AP-AS1 in ccRCC cells. Pro-angiogenic and pro-inflammatory abilities of MCM3AP-AS1 *in vivo* were confirmed in mice subcutaneously xenografted with human ccRCC cells. Our findings demonstrate a novel mechanism by which lncRNA MCM3AP-AS1 exerts pro-angiogenic and pro-inflammatory effects, highlighting the potential of MCM3AP-AS1 as a promising target for treating ccRCC.

## Introduction

Kidney cancer is one of the most prevalent cancers around the world, with almost 270,000 new diagnoses and 116,000 deaths being attributed to the malignancy every year (1). Approximately 90% of these cases are caused by renal cell carcinoma (RCC), a malignancy arising from the renal tubular epithelium (2). Clear cell renal cell carcinoma (ccRCC) is the main subtype of RCC, and its natural history is complicated in nature and extremely difficult to predict (3). Currently, vascular endothelial growth factor (VEGF) and mammalian target of rapamycin (mTOR)-directed therapies represent the standard of care for metastatic ccRCC, while the molecular sub-classifications of ccRCC remain to be fully elucidated (4). Discoveries of a wide range of inter- and intra-tumoral genetic changes support the notion that ccRCC represents a series of molecularly related, but distinct diseases defined by combinatorial genetic variations in tumor cells (5). Accumulating evidences have also shown that various long non-coding RNAs (lncRNAs) exert abnormal expression patterns in ccRCC, and could serve as potential novel molecular biomarkers (6).

LncRNAs have emerged as gene regulators over the last few years, owing to their ability to influence various aspects of cellular homeostasis and genomic stability in several cancers, including kidney cancer (7, 8). Numerous studies have also highlighted the critical value of lncRNAs as prognostic biomarkers for ccRCC (9). For instance, lncRNA NONHSAT123350 participates in the pathogenesis of ccRCC (10). The expression patterns and functions of MCM3AP-AS1 have also been investigated in various cancers. It has been shown that lncRNA MCM3AP-AS1 plays an oncogenic role in hepatocellular carcinoma (11, 12) and papillary thyroid cancer (13). In addition, MCM3AP-AS1 has been proved to be closely-related to the regulation of glioblastoma angiogenesis (14). However, there is limited data in terms of the mechanism of MCM3AP-AS1 mediated angiogenesis in ccRCC.

Recently, E2 promoter binding factor 1 (E2F1) and dipeptidyl peptidase 4 (DPP4) have been predicted as the downstream transcription factors and target genes of MCM3AP-AS1 by the LncMAP database. Moreover, high levels of E2F1 are associated with the development of ccRCC (15). Other studies by several research groups have also reported that over-expression of E2F1 can promote the proliferation, migration and invasion of ccRCC cells (16, 17). Meanwhile, DPP4 is a membrane-bound protein and its soluble form is present in body fluids, existing in various cell types (18). Furthermore, it has been reported that the DPP4 is closely associated with ccRCC. For example, the 5-year survival rates of ccRCC patients with higher activity levels of DPP4 are shorter than those with lower levels (19). Soluble DPP4 activity is considered to be positively-related to the aggressiveness of ccRCC, which was evidenced by the observation that higher activities of DPP4 were detected in high grade ccRCC tumors (20). Herein, the current study aims to investigate the functional association and underlying molecular mechanism of MCM3AP-AS1 and its interactions with transcription factor E2F1 and DPP4 in ccRCC.

## Materials and Methods

### Ethics Approval

The current study was performed with the approval of the Ethics Committee of the Second Hospital of Jilin University (approval code: 201201021). Written informed consents were obtained from participants prior to specimen collection. Animal experimentation in the current study was permitted by the Animal Ethics Committee of the Second Hospital of Jilin University (approval code: 201909056). All animals received humane care according to the *Guide for the Care and Use of Laboratory Animals* published by the US National Institutes of Health, and great efforts were made to minimize the suffering of the included animals (21).

### Tissue Specimen Collection and Cell Culture

Tumor tissues and matched adjacent non-tumor tissues were surgically collected from 78 ccRCC patients at the Second Hospital of Jilin University from February 2012 to December 2013. None of the included patients received anticancer treatment prior to specimen collection. All obtained samples were staged and graded according to the Classification of Tumor Lymph Node Metastasis (TNM) and World Health Organization (WHO) criteria.

Additionally, the human ccRCC cell lines 786-O, Caki-1, UT14, UT48 and human renal tubular epithelial cell line HK-2 (ATCC, Rockville, MD, USA) were grown in a cell culture incubator with 5% CO _2_ in air at 37°C. The cells were then cultured in RPMI-1640 medium (Gibco, Thermo Fisher Scientific, Waltham, MA, USA) containing 10% fetal bovine serum, 100 ug/mL streptomycin and 100 IU/mL penicillin.

### Lentiviral Transduction

The full-length of MCM3AP-AS1 was cloned into a mammalian expression vector pcDNA3.1 (+) (GenePharma, Shanghai, China). Next, shRNA sequences targeting MCM3AP-AS1, E2F1 and DPP4 were designed and cloned into the RNAi expression vector pRNAU-6.1/neo (GenePharma). Human ccRCC cells were then transfected with the aforementioned recombinant plasmids following the instructions of Lipofectamine 3000. Stable knockdown of MCM3AP-AS1 were achieved using the PLKO-Puro plasmid (Sigma Chemical Co., USA) inserted with short hairpin RNA against MCM3AP-AS1 (sh-MCM3AP-AS1) and transduction with lentivirus vectors psPAX2 and pMD2.G (Addgene, Cambridge, MA, USA).

### Real Time Quantitative PCR (RT-qPCR)

Total RNA content was extracted using TRIzol (15596026, Invitrogen, Carlsbad, California, USA). RNA was reverse transcribed into cDNA using a reverse transcription kit (RR047A, Takara Bio Inc., Otsu, Shiga, Japan). The samples were loaded using a SYBR Premix EX Taq kit (RR420A, Takara Bio Inc., Otsu, Shiga, Japan), and subjected to RT-qPCR reaction using a real-time PCR instrument (ABI7500, ABI, Foster City, CA, USA). Primers were synthesized by Shanghai Sangon Biotechnology Co. Ltd. (Shanghai, China) (Table 1). β-actin was used as an internal reference. The relative expression of the product was calculated using the 2^−ΔΔCt^ method.

**Table 1:** Primer sequences for RT-qPCR.

**Gene**	**Primer sequences**
MCM3AP-AS1	F:5′-GCTGCTAATGGCAACACTGA-3′
	R:5′-AGGTGCTGTCTGGTGGAGAT-3′
DPP4	F:5′-CTCCAGAAGACAACCTTGACCATTACAGAA-3′
	R:5′-TCATCATCATCTTGACAGTGCAGTTTTGAG-3′
Ki67	F:5′-CGTCCCAGTGGAAGAGTTGT-3′
	R:5′-CGACCCCGCTCCTTTTGATA-3′
VEGF-A	F:5′-GCTACTGCCATCCAATCGAG-3′
	R:5′-CTCTCCTATGTGCTGGCCTT-3′
TNF-α	F:5′-GAGCACTGAAAGCATGATCC-3′
	R:5′-CGAGAAGATGATCTGACTGCC-3′
IL-1β	F:5′-AAACCTCTTCGAGGCACAAG-3′
	R:5′-GTTTAGGGCCATCAGCTTCA-3′
IL-6	F:5′-GGCACTGGCAGAAAACAACC-3′
	R:5′-GCAAGTCTCCTCATTGAATCC-3′
GAPDH	F:5′- TGACTTCAACAGCGACACCCA−3′
	R:5′- CACCCTGTTGCTGTAGCCAAA−3′

*F, forward; R, reverse*.

### Western Blot Analysis

Total protein content in tissues or cells was extracted using a radio-immunoprecipitation assay (RIPA) lysate containing phenylmethylsulfonyl fluoride (PMSF). The procedures for Western blot were performed as previously described (22), with the following primary antibodies purchased from Abcam Inc. (Cambridge, UK): anti-DPP4 (ab28340, dilution ratio of 1:1,000), anti-Ki67 (ab16667, dilution ratio of 1:1,000), anti-VEGF-A (ab46154, dilution ratio of 1:1,000), anti-GAPDH (ab9485, dilution ratio of 1:2,500), and horseradish peroxidase-conjugated anti-rabbit IgG (ab97051, dilution ratio of 1:2,000, Abcam Inc.). Results were visualized with enhanced chemiluminescence detection reagents. The protein content was represented by the ratio of gray value between the targets and internal reference (GAPDH).

### Methylation-Specific PCR (MSP)

Cells were treated with 5-Aza-2′-deoxycytidine (5-Aza-dC) (Sigma-Aldrich, USA) dissolved in dimethyl sulfoxide (DMSO) for 48 h. The genomic DNA was then manipulated and purified according to the instructions of the TGuide genomic DNA extraction kit (OSR-M401, Tiangen BioTechl (Beijing) Co., Ltd., Beijing, China). After modification by sodium bisulfite, PCR was carried out for a total of 38 cycles (1 min for 95°C, 1 min for 60–63°C, 1 min for 72°C) employing 200 ng of bisulfite-modified genomic DNA as a template. The PCR product (10 μL) was then run on a 2% agarose gel and visualized by detection of ethidium bromide staining.

Under the same conditions, the same samples were subjected to PCR with methylated-specific and unmethylated-specific primers, respectively (Table 2). Images were scanned using a gel electrophoresis imager (Alpha Imager^TM^2200, Alpha Innotech, San Leandro, CA, USA). With β-actin serving as NC, methylated-specific and unmethylated-specific primer bands were photographed and analyzed semi-quantitatively to calculate the optical density (OD) value ratio of the methylated-specific bands to unmethylated-specific bands. The ratio was calculated using the formula as ratio = (methylated OD value/NC OD value)/(unmethylated OD value/NC OD value).

**Table 2:** Primer sequences for methylation-specific PCR.

**MCM3AP-AS1**	**Primer sequences**
Methylated	F: 5′-TTTATTCGTTTTTTTGAGAGAAAAC-3′
	R: 5′-AAACCGACCTAAAAACCTACG-3′
Unmethylated	F: 5′-TATTTGTTTTTTTGAGAGAAAATGT-3′
	R: 5′-AACAAACCAACCTAAAAACCTACAC-3′
beta-actin	F: 5′-AAGATCTGGCACCACACCTTC-3′
	R: 5′-CACACCATCACCAGAATCGA-3′

*F, forward; R, reverse*.

### Fluorescence *in situ* Hybridization (FISH)

The subcellular localization of MCM3AP-AS1 was identified using the FISH technique according to the instructions of RiboTM FISH Probe Mix (Red) (C10920, RiboBio Co., Ltd., Guangzhou, China). Briefly, ccRCC cells were seeded in a 24-well plate at a density of 6 × 10^4^ cells/well. When cell confluence reached 60–70%, 1 mL 4% paraformaldehyde was used to fix the cells at room temperature for 10 min., followed by the addition of 1 mL/well pre-cooled dialysis solution (PBS containing 0.5% Triton X-100) for 5 min at 4°C, and 200 uL/well pre-hybridization at 37°C for 30 min. Next, the cells were added with appropriate amounts of probe hybridization solution containing the probe (anti-MCM3AP-AS1 nucleotide probe, Wuhan GeneCreate Biological Engineering Co., Ltd., Wuhan, China) for hybridization at 37°C in dark conditions. Next, the 4,6-diamino-2-phenyl indole (DAPI) staining solution (1:800) was employed to stain the cells for 10 min. Nail polish was used to mount the cells, which were observed under a fluorescence microscope (Olympus, Tokyo, Japan) with five different fields of view selected for observation and photographing.

### Native RNA Immunoprecipitation (RIP)

The binding of MCM3AP-AS1 to the transcription factor E2F1 protein was detected using RIP kits (Merck Millipore, Billerica, MA, USA). Cells were lysed with equal amounts of lysate for 5 min, and were then centrifuged at 20,000 × g for 10 min at 4°C. Each coprecipitation reaction system was washed with 50 μL of magnetic beads and resuspended in 100 μL of RIP Wash Buffer, followed by incubation with 1 μg of antibody. The magnetic bead-antibody complex was then washed and resuspended in 900 μL of RIP Wash Buffer, and 100 μL of the cell extract was added for incubation at 4°C overnight. Next, the sample was placed on a magnetic stand to collect the magnetic bead-protein complex. The sample was detached with proteinase K to extract RNA for subsequent RT-qPCR. The antibody used for RIP was E2F1 antibody (ab112580, dilution ratio of 1:200, Abcam, Cambridge, UK). An IgG antibody (ab172730, dilution ratio of 1:100, Abcam, Cambridge, UK) was used as NC.

### Cross-Linking RIP

The ccRCC cells transfected for 48 h were cross-linked with 0.75% formaldehyde, and the cells were trypsinized and resuspended in PBS. Nuclei were pelleted by means of centrifugation and resuspended in RIP buffer (150 mM KCl, 25 mM Tris, pH 7.4, 5 mM EDTA, 0.5 mM DTT, 0.5% NP40 and 100 U/mL RNase inhibitor). The chromatin was sheared by sonication, and then the cells were centrifuged to extract the supernatant. The E2F1 antibody (ab112580, dilution ratio of 1: 200, Abcam) was added to the nucleic acid extract, and the cells were incubated overnight at 4°C. Pierce protein A/G Magnetic Beads (88803, Thermo Fisher Scientific, USA) were then added and the cells were further incubated for 4 h at 4°C. The samples were then placed on a magnetic stand to collect the magnetic bead-protein complex. After trypsinization by protein K, the extracted RNA was reverse transcribed into cDNA. The analysis was then carried out using a RT-qPCR experiment.

### RNA Pull-Down Assay

Biotinylated MCM3AP-AS1 and U6 RNA were mixed with proteins obtained from the nuclear extracts of ccRCC cells. The complex of biotinylated MCM3AP-AS1 and protein was isolated using streptavidin agarose beads (Thermo Fisher Scientific, USA). Next, the proteins were eluted from the RNA-protein complex and immunoblotted using E2F1 antibody.

### Chromatin Immunoprecipitation (ChIP)

The ccRCC cells were fixed with formaldehyde for 10 min, and the chromatin fragment (200–1,000 bp) was sonicated. The cells were then centrifuged at 12,000 × g for 10 min at 4°C to collect the supernatant. The NC rabbit IgG (ab172730, dilution ratio of 1: 100, Abcam, Cambridge, UK) and E2F1 antibody (ab112580, dilution ratio of 1: 200, Abcam, Cambridge, UK) were added, respectively, and the cells were then incubated overnight at 4°C. The complex of protein and DNA was precipitated with Pierce protein A/G Magnetic Beads (88803, Thermo Fisher Scientific, USA), and the cells were centrifuged at 12,000 × g for 5 min, while the supernatant was discarded and the non-specific complex was washed away. The cross-linking was carried out overnight at 65°C, and the DNA fragment was recovered by phenol/chloroform extraction purification. An amplified primer was designed to containing site2 binding to E2F1 and DPP4 DNA promoter (F: 5′-CCAAAACCCTTGAAAAGACTATGAG-3′, R: 5′-AAGCAGTGAGGGGTCTAAAGCAGTA-3′), the amplified product was 365 bp long, containing E2F1 and DPP4. The DNA promoter bound to the sequence of site4 (1,873–1,883 bp), 11 bp from the transcription start site. A primer that could amplify the sequence away from the DPP4 DNA promoter region was designed as the NC for the site 4 primer (F: 5′-GACTTTGCTCCTCATTTGTCTTCAG-3′, R: 5′-TCTCTTCCACATTTTTGGGTATCTT-3′), and the Distal primer was expanded. The product was 201 bp long and 1,126 bp from the transcription start site. The recovered DNA fragment was used as the amplification template, and site 4 primers and Distal primers (control) were added respectively to perform rt-qPCR experiments to verify whether site 4 of DPP4 DNA was the site of transcription factor E2F1 binding.

After silencing MCM3AP-AS1, a sample containing the purified DNA fragment was finally obtained for ChIP assay (the same as above), and the cells transfected with sh-NC were used as control. The primers of site 4 were used to detect the change in quantity of the binding of E2F1 antibody at DPP4 promoter site 4.

### Cell Counting Kit-8 (CCK-8) Assay

One day prior to transfection, the cells were seeded into 96-well culture plates at a density of 2 × 10^3^cells/well. After carrying out transfection for 24, 48, and 72 h, about 10 μL of CCK8 (Dojindo Molecular Technologies, Inc., Kyushu, Japan) reagent was added to each well for incubation at 37°C for 2 h. Absorbance values were measured at a wavelength of 450 nm using a microplate reader (Bio-Rad, Hercules, CA, USA).

### 5-Ethynyl-2′-deoxyuridine (EdU) Staining Assay

Cell proliferation was determined by an ethynyl-2-deoxyuridine incorporation assay using EdU Apollo DNA *in vitro* kits (RiboBio Co., Ltd., Guangzhou, China). One day before transfection, 5 × 10^3^ cells/well were seeded in 96-well plates. After 48 h of transfection, 100 μL of 50 μM EdU solution was added to each well for incubation at 37°C for 2 h. The cells were then fixed at room temperature for 30 min using 100 μL of PBS containing 4% polyoxymethylene. Next, the cells were incubated with 50 μL of 2 mg/ml glycine for 5 min. After permeabilizing the cells with 0.5% TritonX, the cells were reacted with 1 × Apollo solution in dark conditions at room temperature for 30 min. DAPI (Sigma-Aldrich Chemical Company, St. Louis, MO, USA) was used to stain nucleus for 10 min. The cells were then observed under an inverted fluorescence microscope and photographed, and the positive cell rate of EdU-positive cells/DAPI-positive cells was counted using the Image-Pro-Plus 6.0 software (Media Cybernetics, USA).

### Human Umbilical Vein Endothelial Cell (HUVEC) Tube Formation Experiment

The 96-well plates were coated with 100 μL Matrigel (BD Biosciences, Bedford, MA, United States) per well and maintained at 37°C for 30 min. Thereafter, HUVEC cells were seeded in the plates at a density of 1 × 10^4^ cells/well. After the cells were fully adhered, the medium was replaced with conditioned medium from Caki-1 and 786-O cells. After 24 h of incubation, the cells were monitored and imaged using an Olympus DP71 immunofluorescence microscope (Olympus, Tokyo, Japan), and the total length and branch number of the tubules in the focused area were measured and analyzed using the Chemi Imager 5500 V2.03 software (Alpha Innotech, San Leandro, CA, United States) (14, 23).

### Enzyme Linked Immunosorbent Assay (ELISA) Methods

The human inflammation-related factors TNF-α, IL-1β, IL-6 were detected using the human TNF-α ELISA kit (ab181421), human IL-1β ELISA kit (ab46052), human IL-6 ELISA kit (ab178013), respectively. All operations were carried out according to the manufacturer's instructions.

### Nude Mouse Tumor Models of Human ccRCC

A total of 20 BALB/C nude mice (aged 4–6 weeks) were randomly divided into two groups, with 10 mice in each group. The ccRCC cells expressing sh-MCM3AP-AS1 expression or the relevant NC were subcutaneously inoculated into the thighs of nude mice. The tumor volume was examined weekly and calculated according to the following formula: (a^*^b^2^)/2 (a was the longest path of the tumor and b was the shortest path of the tumor). The transplanted tumor volume was recorded to plot the growth curve. Finally, the mice were euthanized by carbon dioxide asphyxiation 6 weeks after injection, and the tumors were harvested and weighed.

### Immunohistochemistry

The transplanted tumor tissues were paraffin-embedded and made into 5 μm sections. The slides of tumor sections were immunostained with anti-Ki67 (dilution ratio of 1:100, ab15580, Abcam) and anti-CD34 (dilution ratio of 1:50, ab64480, Abcam). Immunohistochemical staining of CD34 was employed to evaluate microvessel density (MVD) and analyzed by two experienced pathologists according to the principles previously reported by Windner (24).

### Chicken Chorioallantoic Membrane (CAM) Angiogenesis Test

The fertilized eggs were incubated overnight at 37°C with 70% humidity. On day 7, a sterilized filter paper tray (0.5 cm diameter) was placed on the surface of the CAM. And every 3 days, 30 μL of the supernatant from the experimental and control groups was dropped onto the filter paper tray which was sealed with scotch tape. On the 10th day of incubation, the MacroPATH Srl imaging system (Sorisole BG, Italy) was used to take photograph and evaluate the number of blood vessels around the filter paper tray.

### Statistical Analysis

All data are shown as mean ± standard deviation and analyzed using the SPSS 21.0 software (IBM, Armonk, NY, USA), with a value of *p* < 0.05 indicating statistical significance. Paired *t-*test was used for the comparison of two paired sets of data with normal distribution and variance; otherwise, unpaired *t*-test was applied. Comparisons among multiple groups were analyzed by one-way analysis of variance (ANOVA) with Tukey's test or by repeated measures ANOVA with Bonferroni test at different time points. The relationship between MCM3AP-AS1 and DPP4 was analyzed using Pearson correlation. The Kaplan-Meier method was used to calculate the survival rate of patients. Prognostic factors were analyzed by multivariate COX regression analysis.

## Results

### MCM3AP-AS1 Was Associated With the Development and Prognosis of ccRCC

Aiming to screen the possible genes implicated in the development of ccRCC, a differential gene expression analysis was performed on the ccRCC-related GSE15641 dataset retrieved from the GEO database, with 267 differentially expressed genes being selected. Amongst the 267 genes, four lncRNA exhibited significant differences between ccRCC and normal samples (Table 3), of which MCM3AP-AS1 showed the largest change and the most significant difference compared to the normal sample.

**Table 3:** Differential expression analysis of lncRNAs in the GSE15641 dataset.

**ID**	**logFC**	**AveExpr**	**t**	***P*-Value**	**adj. *P*-Val**
MCM3AP-AS1	2.411975442	6.277541763	34.81494166	6.06E-40	2.96E-37
MYCNOS	−2.020677798	6.97198263	-33.58519948	4.24E-39	1.77E-36
LINC00588	−2.226003664	4.742486547	-33.54918328	4.49E-39	1.82E-36
LINC00472	−2.373794553	5.429089954	-16.17437925	6.75E-23	1.92E-21

**Figure 1:**
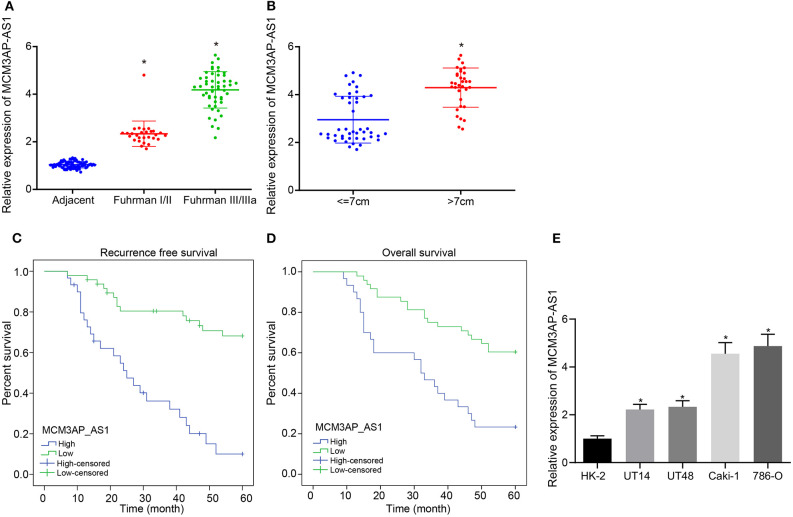
The expression of MCM3AP-AS1 is associated with clinicopathological features of ccRCC patients and the overall survival. **(A)** RT-qPCR assay examined the expression of MCM3AP-AS1 in adjacent tissues and Fuhrman I/II (28 cases) and III/IIIa (50 cases) grade ccRCC tissues, and * (relative to adjacent tissues) stands for *p* < 0.05. **(B)** RT-qPCR assay examined the expression of MCM3AP-AS1 in ccRCC tumors ≤ 7 cm (45 cases) and > 7 cm (33 cases), * *p* < 0.05 vs. tumor size ≤ 7 cm. **(C)** Kaplan-Meier survival curve analyzing the recurrence-free survival time of highly-expressed MCM3AP-AS1 patients and poorly-expressed MCM3AP-AS1 patients. **(D)** Kaplan-Meier survival curve analysis of highly-expressed MCM3AP-AS1 patients and poorly-expressed MCM3AP-AS1 patients with overall survival. **(E)** RT-qPCR assay examined the expression of MCM3AP-AS1 in human normal tubular epithelial cell line HK-2 and four human ccRCC cell lines 786-O, Caki-1, UT14, UT48, * stands for *p* < 0.05, relative to HK-2 cell line. The figures above were all measurement data, expressed as mean ± standard deviation. Paired *t*-test was used for comparison between two groups. Survival rates were measured using Kaplan-Meier methods followed by Log-rank test. Comparisons among multiple groups were analyzed by ANOVA, followed by Tukey's *post hoc* test.

To verify the above results, the expression of MCM3AP-AS1 was determined in matched adjacent non-tumor tissues and Fuhrman I, II, III, and IV ccRCC tissues by RT-qPCR (Figure 1A). The expression of MCM3AP-AS1 was up-regulated in ccRCC tissues relative to adjacent tissues (*p* < 0.05), and the higher the ccRCC tissue grade, the more significant up-regulation of MCM3AP-AS1 was. The expression of MCM3AP-AS1 was then examined in 45 ccRCC tumors ≤ 7 cm and 33 tumors >7 cm using RT-qPCR (Figure 1B). The results showed that the expression of MCM3AP-AS1 in ccRCC tumors > 7 cm was significantly higher than ≤ 7 cm (*p* < 0.05). However, the expression of MCM3AP-AS1 was not correlated with age or gender (Table 4). These findings suggested that elevated expressions of MCM3AP-AS1 might play an important role in the progression of ccRCC. Subsequently, the survival time of ccRCC patients with higher expression (above median value, *n* = 30) and lower expression (below median value, *n* = 48) of MCM3AP-AS1 were compared (Figures 1C,D). The results demonstrated that patients with lower expression of MCM3AP-AS1 exhibited higher recurrence-free survival and overall survival relative to patients with higher MCM3AP-AS1 expressions. In addition, univariate analysis showed that high expression of MCM3AP-AS1 and high Fuhrman grading were both associated with an increased risk of cancer-related death. Next, univariate analysis and prognostic-related factors were included in the COX model for multivariate analysis, and the results revealed that MCM3AP-AS1 was highly expressed (OR = 2.733, *p* = 0.909, 95% CI = 1.290 - 5.790), which was an independent prognostic factor for ccRCC patients (Table 5). RT-qPCR assay was further applied to detect the expression of MCM3AP-AS1 in human normal tubular epithelial cell line HK-2 and four human clear cell carcinoma cell lines 786-O, Caki-1, UT14 and UT48 (Figure 1E). Relative to HK-2 cells, the expression of MCM3AP-AS1 was markedly higher in all the four human ccRCC cell lines (*p* < 0.05), with the most evident increases in Caki-1 and 786-O cells, which were selected for subsequent experiments.

**Table 4:** Clinical data of ccRCC patients and analysis of MCM3AP-AS1 expression in these patients.

**Item**	**Case**	**MCM3AP-AS1 expression**	***p***	
Age (year)	<60	56	3.446 ± 1.17	0.353
	≥60	22	3.711 ± 1.000	
Sex	Male	45	3.652 ± 1.14	0.23
	Female	33	3.341 ± 1.096	
Tumor size (cm)	≤ 7	45	2.954 ± 0.97	0.001
	>7	33	4.293 ± 0.819	
Fuhrman tumor grade	I–II	28	2.338 ± 0.53	0.001
	IIIa	50	4.183 ± 0.767	

**Table 5:** Analysis of independent prognostic factors in patients with ccRCC.

**Variable**	**Univariate**			**Multivariate**		
	**Hazard ratio**	**95% CI**	***P*-value**	**Hazard ratio**	**95% CI**	***P*-value**
Age (<60 vs. ≥ 60)	1.012	0.990–1.035	0.299	–	–	–
Gender (Male vs. Female)	1.143	0.617–2.118	0.67	–	–	–
Tumor size (>7 cm vs. ≤ 7 cm)	0.609	0.332–1.116	0.109	–	–	–
MCM3AP-AS1 expression (High)	3.026	1.638–5.589	0.001	2.733	1.290–5.790	0.009
Fuhrman grade (IIIa)	2.152	1.057–4.380	0.035	1.216	0.509–2.905	0.66

### MCM3AP-AS1 Expression Was Negatively Related to the Degree of Methylation of Its Promoter

**Figure 2:**
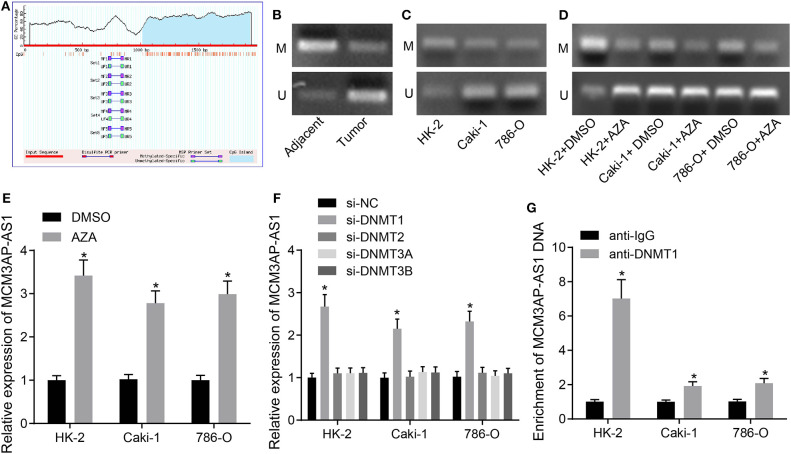
Correlation between the degree of DNA methylation and the expression of MCM3AP-AS1. **(A)** MethPrimer website (http://www.urogene.org/cgi-bin/methprimer/methprimer.cgi) analyzed that LCCRNA MCM3AP-AS1 promoter region was observed in CPG island. **(B,C)** The methylation level of CpG island in LCMRNA MCM3AP-AS1 promoter region was examined in ccRCC tumor tissues and adjacent tissues **(B)** and in Caki-1 and 786-O cells **(C)**. **(D)** The degree of methylation of the MCM3AP-AS1 promoter treated with AZA was examined. **(E)** RT-qPCR assay examined the expression of MCM3AP-AS1 in cells treated with AZA, * (relative to the cells treated with DMSO) stands for *p* < 0.05. **(F)** RT-qPCR assay examined the expression of MCM3AP-AS1 in cells, * (relative to the cells treated with si-NC) stands for *p* < 0.05. **(G)** ChIP assay examined the methyl-transferase enrichment of MCM3AP-AS1 promoter region in HK-2, Caki-1 and 786-O cells. * (relative to the cells treated with anti-IgG) stands for *p* < 0.05. # (relative to the HK-2 cells) stands for *p* < 0.05. The figures above were all measurement data, expressed as mean ± standard deviation. Unpaired *t*-test was used for comparison between two groups. Comparisons among multiple groups were analyzed by ANOVA, followed by Tukey's *post hoc* test. M, methylated; U, unmethylated.

In order to investigate the mechanism behind up-regulated MCM3AP-AS1, the CpG island in the promoter region of MCM3AP-AS1 was identified using the bioinformatics website MethPrimer (Figure 2A). The methylation levels of the CpG island in the promoter region of MCM3AP-AS1 were then examined in ccRCC tissues and adjacent tissues by MSP assay (Figure 2B). The results showed that the methylation levels were decreased in the ccRCC tumor tissues (OD_methylated:unmethylated_: 0.351), when compared with the adjacent normal tissues (OD_methylated:unmethylated_: 4.526; *p* < 0.05). Additionally, the methylation levels were also measured in HK-2, Caki-1 and 786-O cells (Figure 2C), and the results showed a decrease in the methylation level of the CpG island of the MCM3AP-AS1 promoter region in Caki-1 (OD_methylated:unmethylated_: 0.520) and 786-O cells (OD_methylated:unmethylated_: 0.483) in contrast to the HK-2 cells (OD_methylated:unmethylated_: 3.955; *p* < 0.05). Then, HK-2, Caki-1 and 786-O cells were treated with DNA methyltransferase inhibitor AZA (5′-Aza-d CdR), and the changing methylation degree of MCM3AP-AS1 promoter was also detected (Figure 2D). The results showed that the methylation degree of the MCM3AP-AS1 promoter was decreased in all three cells relative to the cells treated with DMSO. Specifically, the ratio of OD value in HK-2 cells treated with DMSO was 3.801, while that in cells treated with AZA was 0.298. Moreover, the ratio of OD value in Caki-1 cells treated with DMSO to AZA was 0.515 and 0.196. The ratio of OD value in 786-O cells treated with DMSO to AZA was 0.476 and 0.229. Furthermore, the expression of MCM3AP-AS1 was detected in AZA-treated cells by RT-qPCR (Figure 2E), and the results showed that in HK-2, 786-O and Caki-1 cells, the expression of MCM3AP-AS1 in the cells treated with AZA was higher than that of the cells treated with DMSO (*p* < 0.05). Especially, when compared with HK-2 cells, the expression of MCM3AP-AS1 was prominently elevated in 786-O and Caki-1 cells (*p* < 0.05). Next, in order to analyze which DNA methyltransferase was associated with the expression of MCM3AP-AS1, HK-2, Caki-1, and 786-O cells were transfected with plasmids of siRNAs targeting DNMT1, DNMT2, DNMT3A and DNMT3B, respectively. DNMT1 was found to significantly promote the expression of MCM3AP-AS1 (Figure 2F). In addition, ChIP assay was applied to detect the enrichment of MCM3AP-AS1 promoter region methyltransferase (DNMT1) in HK-2, Caki-1 and 786-O cells (Figure 2G). The results showed that compared with the cells treated with anti-IgG, DNMT1 and MCM3AP-AS1 promoter region increased CpG island binding in HK-2 cells treated with anti-DNMT1. Compared with HK-2 cells, Caki-1, and 786-O cells and the cells treated with anti-IgG reduced the binding of DNMT1 with anti-IgG to the CpG island of the MCM3AP-AS1 promoter region (*p* < 0.05). These findings confirmed that MCM3AP-AS1 expression was negatively-related to the degree of methylation of its promoter.

### Silencing of MCM3AP-AS1 Curbed Tumor-Associated Inflammation and Angiogenesis in ccRCC *in vitro*

**Figure 3:**
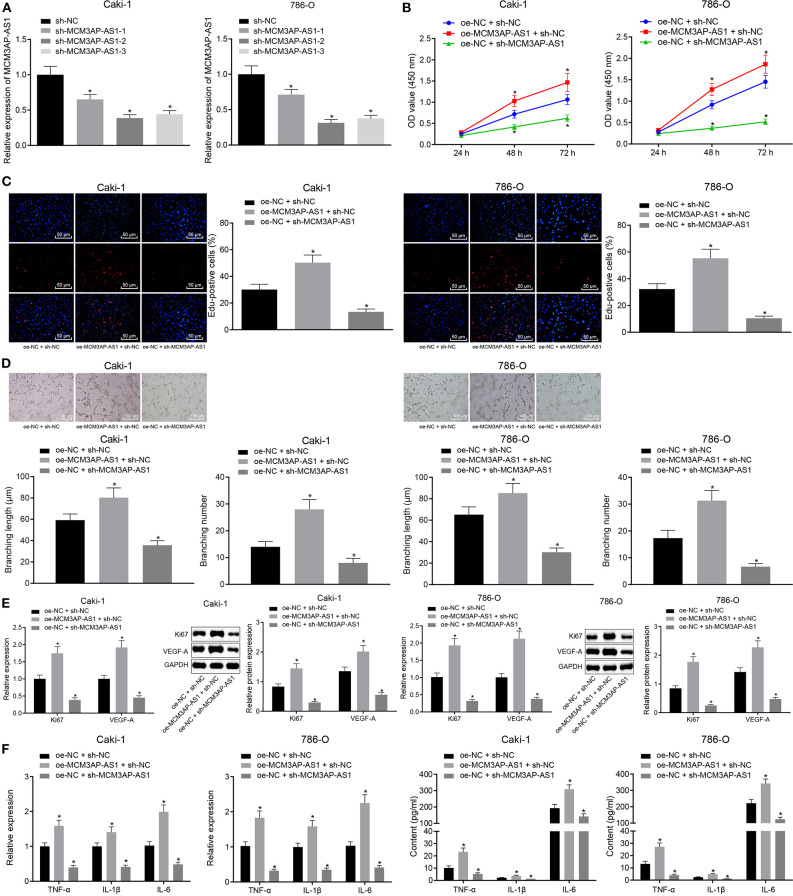
Silencing of MCM3AP-AS1 curbed the release of pro-inflammatory factors and microtube-forming ability of HUVECs in ccRCC cells Caki-1 and 786-O. **(A)** RT-qPCR examined the silencing efficiency of the three sh-MCM3AP-AS1 plasmids. **(B)** CCK-8 method examined cell proliferation. **(C)** EdU assay examined cell proliferation after oe-MCM3AP-AS1 or sh-MCM3AP-AS1 treatment (200 ×). **(D)** Tube formation assay examined cell tube formation ability after oe-MCM3AP-AS1 or sh-MCM3AP-AS1 treatment (100 μm). **(E)** RT-qPCR and Western blot examined the expression of Ki67 and VEGF-A in cells after oe-MCM3AP-AS1 or sh-MCM3AP-AS1 treatment. **(F)** RT-qPCR examined the relative expression of mRNAs of inflammation-related factors TNF-α, IL-1β, and IL-6 in cells after oe-MCM3AP-AS1 or sh-MCM3AP-AS1 treatment. **(G)** ELISA examined the relative expression of TNF-α, IL-1β and IL-6 in the cells after oe-MCM3AP-AS1 or sh-MCM3AP-AS1 treatment. The figures above were all measurement data, expressed as mean ± standard deviation. Unpaired *t-*test was used for comparison between two groups. Comparisons among multiple groups were analyzed by ANOVA, followed by Tukey's *post hoc* test. Data comparison between groups at different time points was performed by repeated measures ANOVA, and Bonferroni was used for *post hoc* testing. In the figure **A** * (relative to the cells infected with sh-NC) stands for *p* < 0.05. In other figures * (relative to the cells infected oe-NC and sh-NC) stands for *p* < 0.05.

To investigate the effect of lncRNA MCM3AP-AS1 on ccRCC cells, three lncRNA MCM3AP-AS1 plasmids (MCM3AP-AS1-1, MCM3AP-AS1-2 and MCM3AP-AS1-3) were designed. Caki-1 and 786-O cells were then transfected with these MCM3AP-AS1 plasmids to silence the MCM3AP-AS1 expression. RT-qPCR results revealed that sh-MCM3AP-AS1-2 brought about the most significant silencing effect (Figure 3A), and therefore, sh-MCM3AP-AS1-2 was selected for subsequent experiments.

Subsequently, Caki-1 and 786-O cells were transfected with over-expression MCM3AP-AS1 vector (oe-MCM3AP-AS1), sh-MCM3AP-AS1 and/or the corresponding NC (oe-NC and sh-NC). The results showed that over-expression of MCM3AP-AS1 promoted the proliferation of Caki-1 and 786-O cells, while silencing MCM3AP-AS1 distinctly inhibited the proliferation of Caki-1 and 786-O cells (Figures 3B,C). Also, over-expressed MCM3AP-AS1 accelerated tube formation in HUVEC cells, yet knockdown of MCM3AP-AS1 reversed the above-mentioned effects (*p* < 0.05) (Figure 3D), Moreover, the expressions of cell proliferation factor Ki67, vascular endothelial growth factor-A (VEGF-A) (Figure 3E) and the inflammation-related factors TNF-α, IL-1β, IL-6 (Figures 3F,G) were found to be up-regulated as a result of MCM3AP-AS1 overexpression. Meanwhile, the expressions of Ki67, VEGF-A, TNF-α, IL-1β, and IL-6 were reduced by MCM3AP-AS1 silencing. These results suggested that overexpression of MCM3AP-AS1 promoted the proliferation, angiogenesis and inflammatory responses of ccRCC cells.

### MCM3AP-AS1 Up-Regulated DPP4 by Entrapment of E2F1 Into the Promoter of DPP4 Gene

**Figure 4:**
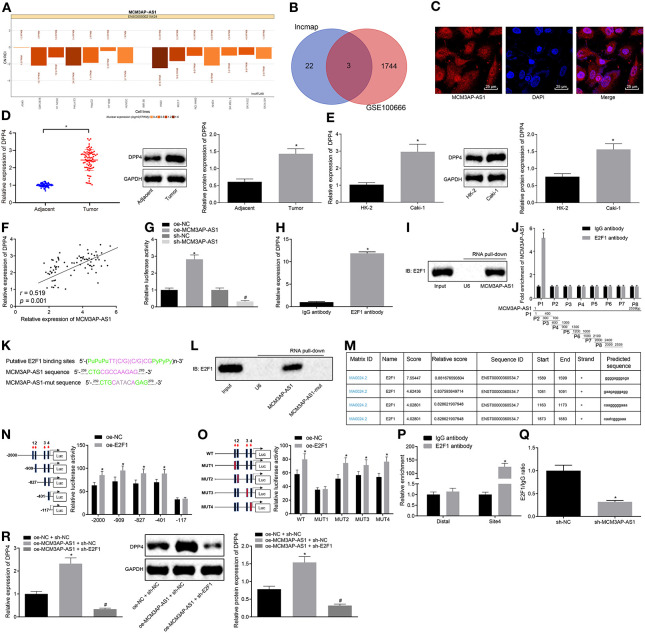
MCM3AP-AS1 recruits E2F1 into the promoter region of the DPP4 gene and transcriptionally activates DPP4. **(A)** Prediction of MCM3AP-AS1 expression cellular location, the histogram below 0 indicating that it is mainly expressed in the nucleus. **(B)** Prediction of downstream target genes of transcription factors. **(C)** RNA-FISH examined the expression of MCM3AP-AS1 in Caki-1 cells (400 ×). **(D)** RT-qPCR and Western blot examined the expression of DPP4 in ccRCC tumor tissues, * (relative to adjacent tissues) stands for *p* < 0.05. **(E)** RT-qPCR and Western blot examined the expression of DPP4 in Caki-1 cells, * (relative to HK-2 cells) stands for *p* < 0.05. **(F)** Pearson correlation analyzed MCM3AP-AS1 and DPP4 expression in 78 ccRCC tumor tissues. **(G)** Dual luciferase reporter assay examined the effect of MCM3AP-AS1 on DPP4 promoter activity, * (relative to the cells infected with oe-NC) stands for *p* < 0.05, # (relative to the cells infected with sh-NC) stands for *p* < 0.05. **(H)** RIP experiments verified that MCM3AP-AS1could bind to transcription factor E2F1, * (relative to the cells treated with IgG antibody) stands for *p* < 0.05. **(I)** RNA pull-down assay verified that transcription factor E2F1 could bind to MCM3AP-AS1. **(J)** Formaldehyde cross-linking RIP assay examined the binding of E2F1 protein to a specific fragment of MCM3AP-AS1. **(K)** Predicted map of specific binding sites for E2F1 and MCM3AP-AS1. **(L)** RNA pull-down assay was performed Caki-1 cells to detect the binding of transcription factor E2F1 to mut-MCM3AP-AS1. **(M)** The website analysis found that the transcription factor E2F1 was most likely to bind to four sites in the DPP4 DNA promoter region. **(N)** Dual luciferase reporter assay examined a truncated DPP4 recombinant luciferase reporter vector and E2F1 expression vector were co-transfected into Caki-1 cells, * (relative to the cells infected with oe-NC) stands for *p* < 0.05. **(O)** The constructed DPP4 recombinant luciferase reporter vector and the E2F1 expression vector were co-transfected into Caki-1 cells for dual luciferase reporter assay, * (relative to the cells infected with oe-NC) stands for *p* < 0.05. **(P)** ChIP assay examined the binding capacity of E2F1 at binding site 4 of the DPP4 promoter region, * (relative to the cells treated with IgG antibody stands for *p* < 0.05. **(Q)** ChIP assay examined the enrichment of DPP4 after silencing MCM3AP-AS1 in Caki-1 cells, * (relative to the cells infected with sh-NC) stands for *p* < 0.05. **(R)** RT-qPCR and Western blot examined the expression of DPP4 in cells, * (relative to the cells infected with oe-NC and sh-NC) stands for *p* < 0.05, # (relative to the cells infected with MCM3AP-AS1 and sh-NC) stands for *p* < 0.05. The values in the figure were measurement data, which are expressed as mean ± standard deviation. Paired design data were compared using paired *t*-test; unpaired *t*-test was used for comparison between groups. Pearson correlation analysis was used to analyze the correlation between MCM3AP-AS1 and DPP4. Data comparisons between groups were performed using one-way ANOVA, followed by Tukey's *post hoc* test.

The lncATLAS database was applied to predict the cellular location of MCM3AP-AS1 expression (Figure 4A), which indicated that MCM3AP-AS1 was primarily expressed in the nucleus. This implied that MCM3AP-AS1 might play some roles in the regulation of other genes through means of transcription factors. In addition, differential analysis of the GSE100666 dataset revealed a total of 1,747 genes that were highly-expressed in ccRCC. The predicted results and microarray analysis results were intersected (Figure 4B), and three genes (DPP4, TICRR, ORC6) were found to be located at the intersection of the two sets of data. Further quantitative analysis of the expression levels of these three genes revealed that the expression of DPP4 in ccRCC showed the most significant change. Further, the LncMAP database was applied to predict the downstream transcription factors and target genes of MCM3AP-AS1, and 25 most downstream target genes were subsequently obtained. Notably, it was found that MCM3AP-AS1 could regulate the expression of DPP4 through the transcription factor E2F1.

The expression of MCM3AP-AS1 in Caki-1 cells was then detected (Figure 4C), which confirmed that MCM3AP-AS1 was indeed mainly distributed in the nucleus. RT-qPCR and Western blot results further revealed that the expression of DPP4 was higher in ccRCC tumor tissues or Caki-1 cells (*p* < 0.05) at both mRNA and protein levels (Figures 4D,E). Also, the correlation analysis of MCM3AP-AS1 and DPP4 expression was performed in 78 ccRCC tumor tissues (Figure 4F), and MCM3AP-AS1 and DPP4 were found to be positively-correlated in ccRCC tumor tissues (*r* = 0.519, *p* = 0.001).

Next, dual luciferase reporter assay was carried out to examine the effect of MCM3AP-AS1 on DPP4 promoter activity (Figure 4G). The results showed that cells infected with oe-MCM3AP-AS1 presented with higher DPP4 promoter activity compared to cells infected with oe-NC (*p* < 0.05). Meanwhile, DPP4 promoter activity in cells infected with sh-MCM3AP-AS1 was lower than that in cells infected with sh-NC (*p* < 0.05). This indicated that MCM3AP-AS1 could positively regulate the expression of the DPP4 gene. RIP assay was applied to determine if MCM3AP-AS1 can bind to E2F1 (Figure 4H). The results showed that there was significantly more MCM3AP-AS1 binding to E2F1 than to IgG (*p* < 0.05), which suggested that MCM3AP-AS1 specifically bound to the E2F1 protein. In addition, RNA pull-down assay further verified that the E2F1 protein specifically bound to MCM3AP-AS1 (Figure 4I). Next, in order to elucidate the specific site of E2F1 protein binding to MCM3AP-AS1, eight pairs of primers were designed to detect different fragments of MCM3AP-AS1. The specific site of E2F1 protein binding to MCM3AP-AS1 was found by formaldehyde cross-linking RIP assay, which was located at the range of 1–400 nt (Figure 4J). Through further analysis, the motif of the transcription factor E2F1 binding RNA was predicted using the JASPAR website. By searching the sequence of MCM3AP-AS1, it was found that its 262–267 nt may bind to the transcription factor E2F1 motif (Figure 4K). Moreover, the binding of the transcription factor E2F1 to mut-MCM3AP-AS1 was detected by mutating the site of MCM3AP-AS1 at 262–267 nt and then performing RNA pull-down assay in Caki-1 cells (Figure 4L). This revealed that the transcription factor E2F1 could not bind to MCM3AP-AS1 after mutating the site of MCM3AP-AS1 at 262–267 nt. Thus, the 262–267nt site (CGCCAA) in MCM3AP-AS1 was indicated as a specific site for binding to the transcription factor E2F1. In order to study the specific site of the E2F1 protein binding to the DPP4 DNA promoter region, the two sites of UCSC and JASPAR were analyzed, which revealed that the E2F1 protein was most likely to bind to the two sites in the DPP4 promoter region (Figure 4M). Dual luciferase reporter assay (Figures 4N,O) was then performed by constructing a truncated or mutated DPP4 recombinant luciferase reporter vector and E2F1 expression vector co-transfected into Caki-1 cells. The results showed that site 4 (CAATCGGGAAA) was the specific site of the E2F1 protein binding to the DPP4 promoter region. Next, the binding ability of E2F1 at the binding site 4 of the DPP4 promoter region was examined in Caki-1 cells by ChIP assay (Figure 4P). The results showed that the amount of amplification product obtained from the site 4 primer in the cells treated with E2F1 was greater than that of the Distal primer in the cells treated with IgG (*p* < 0.05), while there were no significant differences in the amounts in the cells treated with IgG (*p* < 0.05). This indicated that site 4 of the DPP4 promoter region was indeed the site of transcription factor E2F1 binding. To verify the important role of MCM3AP-AS1 in the regulation of both transcription factors E2F1 and DPP4 DNA, ChIP assay was performed after silencing MCM3AP-AS1 in Caki-1 cells (Figure 4Q). The results revealed that the amplified product obtained by RT-qPCR using DPP4 site4 primer was significantly reduced in the sample obtained by enriching the E2F1 antibody of the cells infected with sh-MCM3AP-AS1 relative to the cells infected with sh-NC (*p* < 0.05).

Moreover, in order to further investigate if MCM3AP-AS1 regulates the expression of the DPP4 gene by recruiting the binding transcription factor E2F1, the cells were infected with oe-NC and sh-NC, oe-MCM3AP-AS1 and sh-NC or oe-MCM3AP-AS1 and sh-E2F1, respectively. Subsequent RT-qPCR and Western blot results showed that DPP4 was expressed at higher levels in the cells infected with oe-MCM3AP-AS1 and sh-NC than those infected with oe-NC and sh-NC (*p* < 0.05) (Figure 4R). Cells infected with oe-MCM3AP-AS1 and sh-E2F1, and the cells infected with oe-MCM3AP-AS1 and sh-NC showed decreased DPP4 expressions (*p* < 0.05). It was concluded MCM3AP-AS1 regulated the expression of the DPP4 gene by recruiting the binding transcription factor E2F1.

### Pro-Angiogenic and Pro-Inflammatory Regulation by MCM3AP-AS1 in ccRCC Was Achieved by Regulating DPP4

**Figure 5:**
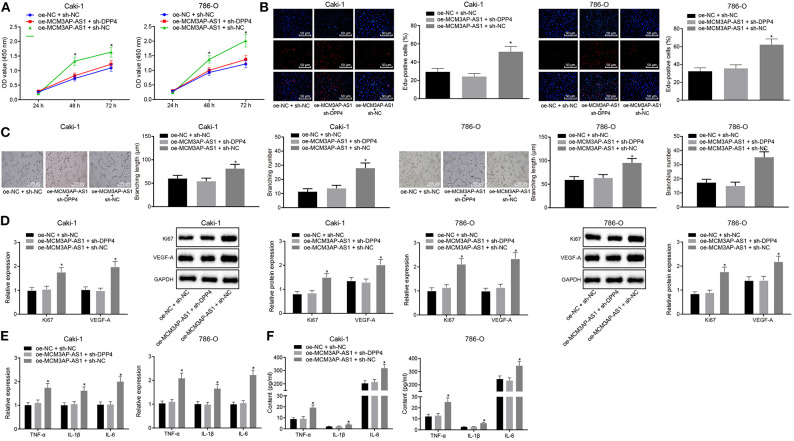
MCM3AP-AS1 promoted tumor inflammation and angiogenesis through regulating DPP4 in ccRCC. **(A)** CCK-8 method examined cell proliferation after oe-MCM3AP-AS1 or sh-DPP4 treatment. **(B)** EdU test examined cell proliferation after oe-MCM3AP-AS1 or sh-DPP4 treatment (200 ×). **(C)** Tube formation test detected tube formation capacity after oe-MCM3AP-AS1 or sh-DPP4 treatment (100 μm). **(D)** RT-qPCR and Western blot examined the expression of Ki67 and VEGF-A in cells after oe-MCM3AP-AS1 or sh-DPP4 treatment. **(E)** RT-qPCR examined the relative levels of mRNAs of inflammation-related factors TNF-α, IL-1β, and IL-6 in cells after oe-MCM3AP-AS1 or sh-DPP4 treatment. **(F)** ELISA examined the relative levels of proteins related to inflammation-related factors TNF-α, IL-1β, and IL-6 after oe-MCM3AP-AS1 or sh-DPP4 treatment. The values in the figure were measurement data, expressed as mean ± standard deviation, * (relative to the Caki-1 and 786-O cells transfected with oe-NC and sh-NC or the Caki-1 and 786-O cells transfected with oe-MCM3AP-AS1 and sh-DPP4) stands for *p* < 0.05. Data comparison between groups at different time points was performed by repeated measures ANOVA, and Bonferroni was used for *post hoc* testing. Comparisons among multiple groups were analyzed by ANOVA, followed by Tukey's *post hoc* test.

In order to explore the effect of MCM3AP-AS1 on the biological characteristics of ccRCC cells by regulating DPP4, Caki-1 and 786-O cells were co-transfected with oe-NC and sh-NC, oe-MCM3AP-AS1 and sh-NC, oe-MCM3AP-AS1 and sh-DPP4, respectively. As is shown in (Figures 5A,B), the proliferation of the Caki-1 and 786-O cells was increased remarkably by transfection of oe-MCM3AP-AS1 and sh-NC, compared with that by oe-NC and sh-NC or oe-MCM3AP-AS1 and sh-DPP4 (*p* < 0.05). The HUVEC tube formation assay results demonstrated that oe-MCM3AP-AS1 and sh-NC significantly enhanced the tube formation ability of HUVEC cells compared to the oe-NC and sh-NC or oe-MCM3AP-AS1 and sh-DPP4 (*p* < 0.05) (Figure 5C). Furthermore, RT-qPCR and Western blot results showed that compared with the Caki-1 and 786-O cells co-transfected with oe-NC and sh-NC or co-transfected with oe-MCM3AP-AS1 and sh-DPP4, the expressions of Ki67 and VEGF-A in the Caki-1 and 786-O cells were significantly up-regulated following transfection with oe-MCM3AP-AS1 and sh-NC (*p* < 0.05) (Figure 5D). Finally, the relative expressions of some inflammation-related factors were detected by RT-qPCR and ELISA (Figures 5E,F), which revealed that relative to oe-NC and sh-NC or oe-MCM3AP-AS1 and sh-DPP4 transfection, oe-MCM3AP-AS1 and sh-NC treatment dramatically up-regulated the expressions of TNF-α, IL-1β, IL-6. These findings revealed that MCM3AP-AS1 exerted pro-angiogenic and pro-inflammatory roles in ccRCC by regulating DPP4.

### Knockdown of MCM3AP-AS1 Inhibited Tumorigenicity, Tumor-Associated Inflammation, and Angiogenesis in ccRCC *in vivo*

**Figure 6:**
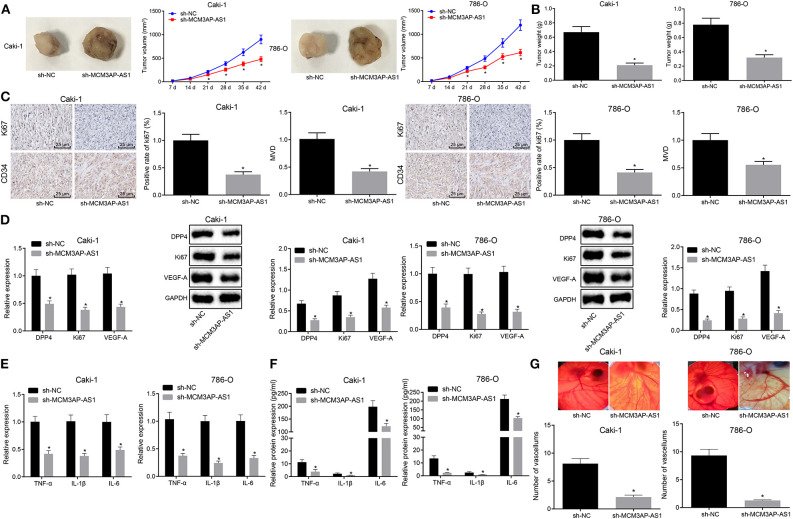
Down-regulation of MCM3AP-AS1 inhibited tumorigenesis, tumor-promoting inflammation and angiogenesis in ccRCC *in vivo*. **(A)** Visual shape and tumor volume of transplanted tumors of nude mice in each group (*n* = 10). **(B)** Weight of transplanted tumors of nude mice in each group (*n* = 10). **(C)** Immunohistochemistry examined the expression of Ki67 in the xenografted tumor tissues of each group and the MVD was calculated by detecting CD34, 400 × (*n* = 10). **(D)** RT-qPCR and Western blot examining the expression of DPP4, Ki67 and VEGF-A in nude mice xenografts (*n* = 10). **(E)** RT-qPCR examined the relative levels of mRNA-related factors TNF-α, IL-1β and IL-6 in nude mice xenografts (*n* = 10). **(F)** ELISA examined the relative levels of inflammation-related factors TNF-α, IL-1β, and IL-6 in nude mice xenografts (*n* = 10). **(G)** CAM angiogenesis assay examined the angiogenesis ability of each group of ccRCC cells. The values in the figure were measurement data, expressed as mean ± standard deviation, * (relative to the mice injected with the cells transfected with sh-NC) stands for *p* < 0.05. Data comparison between groups at different time points was performed by repeated ANOVA, and Bonferroni was used for *post hoc* testing. Comparisons among multiple groups were analyzed by ANOVA, followed by Tukey's *post hoc* test.

In order to verify the conclusions obtained in *in vitro* experiments, Caki-1 and 786-O cells expressing sh-NC or sh-MCM3AP-AS1 were injected into nude mice to analyze tumor formation, and the volume and weight of transplanted tumors were measured and recorded. As is shown in (Figures 6A,B), the mean volume and the weight of the tumor in mice injected with the sh-MCM3AP-AS1-transduced cells were significantly less compared to the mice injected with cells transfected with sh-NC (*p* < 0.05). The expression of Ki67 in the xenografted tumor tissues was further determined by immunohistochemistry and the MVD was calculated by detecting CD34 (Figure 6C). The results revealed that relative to the mice injected with the cells transfected with sh-NC, the expression of Ki67 and the MVD were both decreased in mice injected with cells transfected with sh-MCM3AP-AS1. Moreover, when MCM3AP-AS was silenced, the expressions of DPP4, Ki67 and VEGF-A (Figure 6D), and the inflammation-related factors TNF-α, IL-1β, IL-6 were all remarkably lower (*p* < 0.05) in nude mice transplanted with sh-MCM3AP-AS-transfected cells than that with sh-NC transfected cells (Figures 6E,F). Finally, CAM angiogenesis experiments verified that knockdown of MCM3AP-AS1 remarkably suppressed the angiogenesis of ccRCC cells (Figure 6G). These results demonstrated that down-regulation of MCM3AP-AS1 inhibited ccRCC *in vivo*.

## Discussion

ccRCC remains the most prevalently diagnosed subtype and is more aggressive than papillary and chromophobe RCC (25). As one complex disease, ccRCC only exhibits partial response to current treatment regimens, in addition to a lack of efficacious clinical parameters to gauge the progression and survival of disease (26). Nowadays, strategies such as vascular endothelial growth factor-targeted therapy and mammalian target of rapamycin inhibition are regarded as choice options for treating ccRCC (27). However, the underlying mechanism of ccRCC metastasis still remains unclear (28). Therefore, it is urgent to explore its underlying mechanism using novel approaches to better tackle this malignancy. The results uncovered in the current study suggested that MCM3AP-AS1 promoted tumor inflammation and angiogenesis of ccRCC by regulating DPP4.

Initial findings in our study demonstrated that MCM3AP-AS1 was up-regulated in ccRCC, and associated with poor prognosis in patients with ccRCC. Consistently, previous evidence has also highlighted the use of lncRNAs as a potential prognostic tool to predict the survival of patients with Stage I-III ccRCC (9). MCM3AP-AS1 has been recognized as a versatile mediator in various cancers (13). Moreover, we also found that the expression of MCM3AP-AS1 was negatively related to the degree of methylation of its promoter, as evidenced by markedly lower methylation levels of CpG island in the promoter region of MCM3AP-AS1 in ccRCC tumor tissues relative to adjacent tissues. In addition, over-expression of MCM3AP-AS1 significantly augmented the proliferation, tube formation ability of Caki-1 and 786-O cells and the expressions of Ki67, VEGF-A, TNF-α, IL-1β, and IL-6 in ccRCC Caki-1 and 786-O cells, while silencing MCM3AP-AS1 brought about the opposite results. Consistent with our findings, inhibited expression of the VEGFA gene is correlated with reduction in the time to ccRCC metastasis appearance (29). A previous study also noted that diminished VEGFA suppressed the proliferation, migration and invasion of ccRCC 786-O cells, while promoting their apoptosis (30). Meanwhile, Ki67 is known to function as a critical independent predictor of inferior oncological outcomes in ccRCC patients (31). Similarly, higher levels of TNF-α are often related with tumor progression, whereas anti-TNF-α therapy is commonly administered to RCC patients (32). Furthermore, high expressions of activated inflammasomes, IL-1β and IL-18, are also used as independent predictors of poor prognoses in ccRCC patients, while the tumor promoting cytokine, IL-6, has been implicated in the progression of RCC (33, 34). All these findings and evidence support that MCM3AP-AS1 promoted tumor inflammation and angiogenesis of ccRCC.

Transcription factor, E2F1, is involved in the regulation of cell cycle and cell apoptosis (35). E2F1 has also been demonstrated to contribute significantly to the proliferation, migration and invasion of ccRCC cell *in vitro* (17). In this experiment, the results presented that MCM3AP-AS1 up-regulated the expression of DPP4 by recruiting the transcription factor E2F1 in ccRCC cells. In accordance with our findings, DPP4 has been previously shown to enhance the activity of E2F1 induced by EGF (36). Meanwhile, E2F1 functioning via the signaling mechanism of KLF6-E2F1 axis can promote invasion and metastasis in human ccRCC (15). Furthermore, we also documented elevated levels of DPP4 in ccRCC, wherein MCM3AP-AS1 promoted tumor inflammation and angiogenesis of ccRCC by upregulating DPP4. This is particularly noteworthy as higher activity levels of DPP4 have been related with lower 5-year survival rates in ccRCC patients (19). In addition, soluble DPP4 activity was previously shown to be positively-related with the degree of ccRCC aggressiveness, with higher DPP4 activity correlating with high-grade tumors (20). Inhibition of DPP4 also exerts a crucial role in endothelial growth and may serve as a recovery tool for diabetic vascular complications (37). In addition to treating type 2 diabetes, DPP4 is reported to improve the function of endothelial cells (38). These findings indicate that MCM3AP-AS1 and DPP4 might be involved in the prognosis of ccRCC by interacting with E2F1.

**Figure 7:**
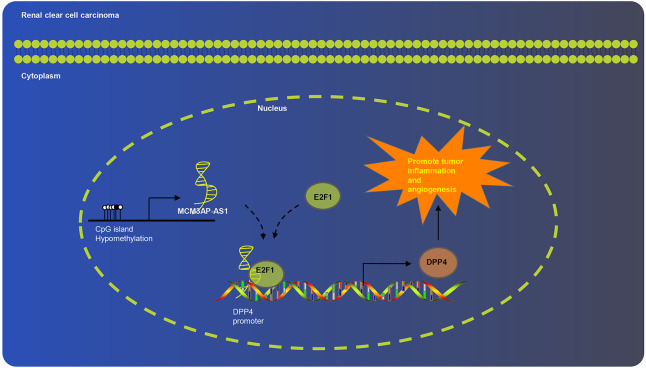
Schematic diagram of MCM3AP-AS1 involved in ccRCC progression. MCM3AP-AS1 promoted tumor inflammation and angiogenesis of ccRCC by regulating DPP4.

Overall, the current study proved that MCM3AP-AS1 promoted tumor inflammation and angiogenesis of ccRCC by regulating DPP4 (Figure 7). It is plausible to suggest that the knockdown of MCM3AP-AS1 impaired inflammation and tumorigenesis in ccRCC. Our findings highlight the MCM3AP-AS1 knockdown-based molecular mechanism as a potential therapy for ccRCC. We recognize that additional mechanisms maybe in place to regulate such pathways, which warrants further exploration to fully realize the therapeutic effect of MCM3AP-AS1.

## Data Availability Statement

All datasets generated for this study are included in the article/supplementary material.

## Ethics Statement

The studies involving human participants were reviewed and approved by the Second Hospital of Jilin University. The patients/participants provided their written informed consent to participate in this study. The animal study was reviewed and approved by the Second Hospital of Jilin University.

## Author Contributions

LQ, YM, and YY designed the study. LQ, XR, and DW collated the data, carried out data analyses, and produced the initial draft of the manuscript. YM and XJ contributed to drafting the manuscript. All authors have read and approved the final submitted manuscript.

## Conflict of Interest

The authors declare that the research was conducted in the absence of any commercial or financial relationships that could be construed as a potential conflict of interest.
